# Supplementary material for: Deep neural networks allow expert-level brain meningioma segmentation and present potential for improvement of clinical practice
Source: Sci Rep. 2022 Sep 14;12:15462. doi: 10.1038/s41598-022-19356-5 (PMC9474556; doi:10.1038/s41598-022-19356-5)
Supplement: Supplementary file 1 — Supplementary Legends. [file 41598_2022_19356_MOESM1_ESM.docx]

**Supplementary Fig. 1. Deep Neural Network Architecture**. The architecture of our deep neural network consisted of a 3D-UNet structured with an encoder and a decoder arm. The network accepts 3D MRI data as input and outputs a 3D segmentation map.

**Supplementary Fig. 2. Tumor location distribution**. Bar plot showing tumor location distribution (% of the total) in the whole meningioma dataset and test set. All the main anatomical locations are adequately represented both in the general dataset and in the test set.

**Supplementary Fig. 3. Tumor volume distribution.** *Left.* Histogram showing the volume distribution of meningiomas in the training set. *Right*. Histogram showing the volume distribution of meningiomas in the test set.

**Supplementary Fig. 4. Current clinical workflow and clinical workflow implementing automatic tumor segmentation**. *Red light*: Volumetric tumor mask is not available; *Green light*: Volumetric tumor mask is available. Above: Volumetric tumor segmentation is currently implemented manually and only for surgical planning purpose, while tumor shape and volume information are not routinely available. Below: The implementation of an automated, expert-level tumor segmentation algorithm provides a tumor mask with accurate shape and volume information at each point of patient care, from the day of the first MRI scan in the radiology suite to the operating room, to each radiologic and clinical follow-up patient encounter.
