# Supplementary figures and images for: Deep neural networks allow expert-level brain meningioma segmentation and present potential for improvement of clinical practice
Source: Sci Rep. 2022 Sep 14;12:15462. doi: 10.1038/s41598-022-19356-5 (PMC9474556; doi:10.1038/s41598-022-19356-5)

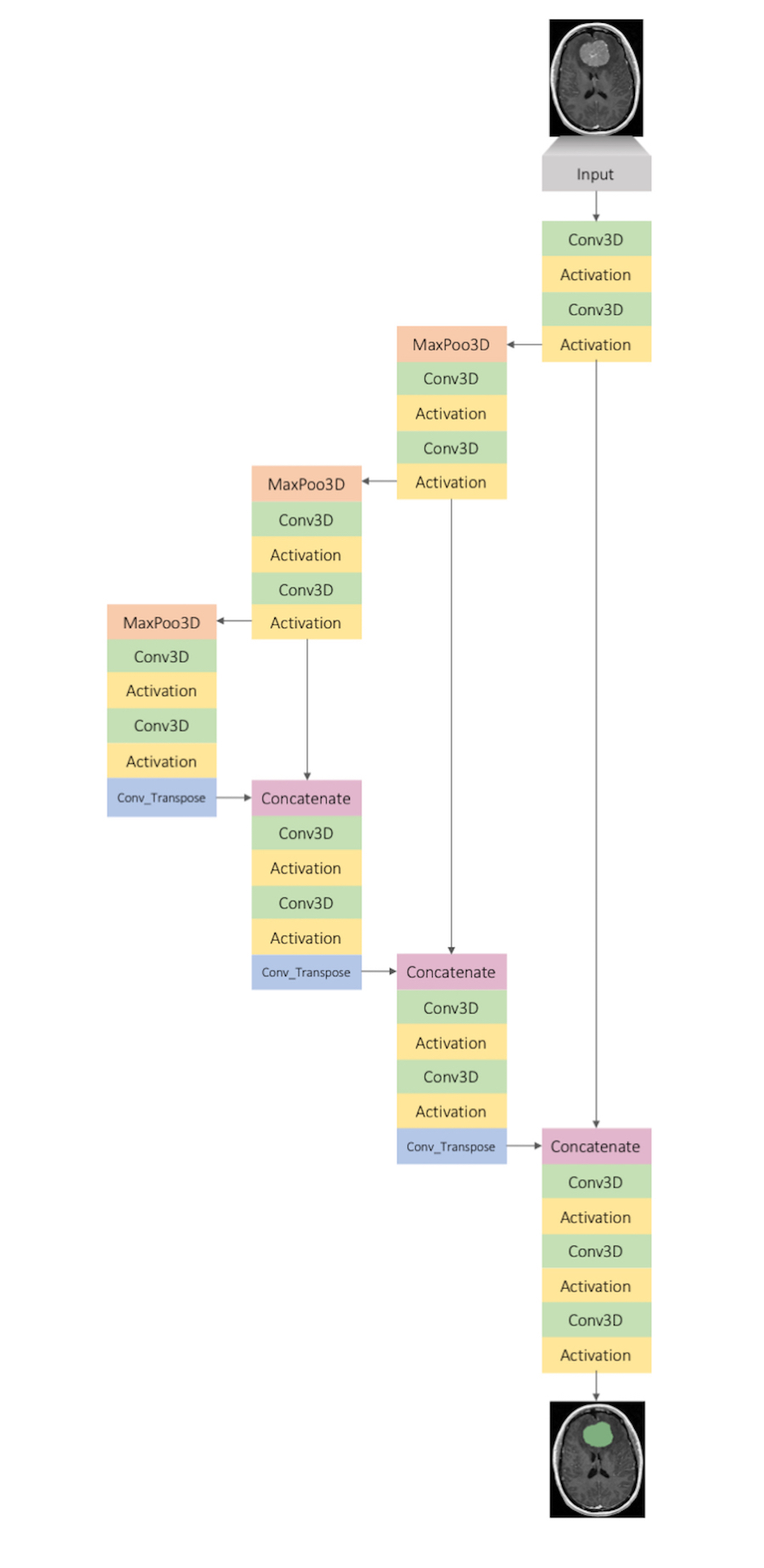

Supplement: Supplementary file 2 — Supplementary Figure 1. [file 41598_2022_19356_MOESM2_ESM.jpg]

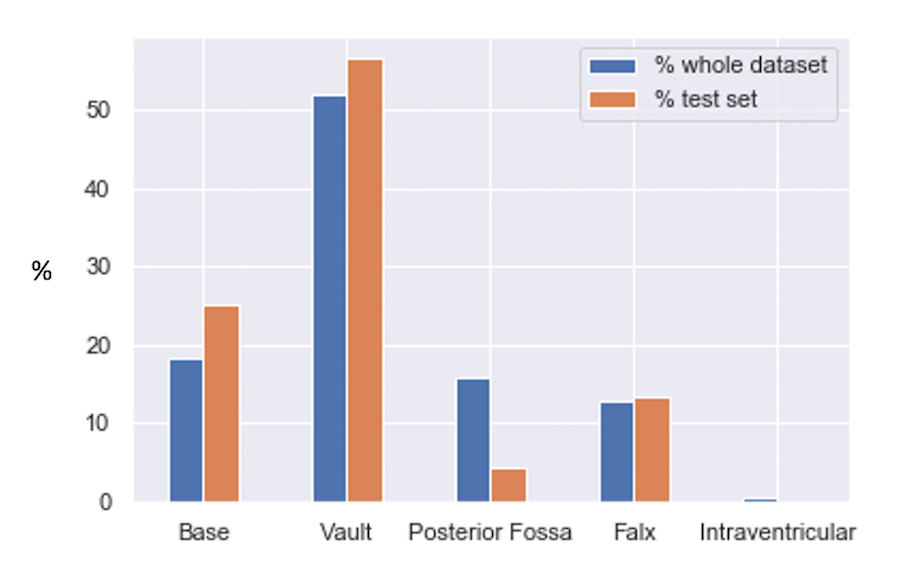

Supplement: Supplementary file 3 — Supplementary Figure 2. [file 41598_2022_19356_MOESM3_ESM.jpg]

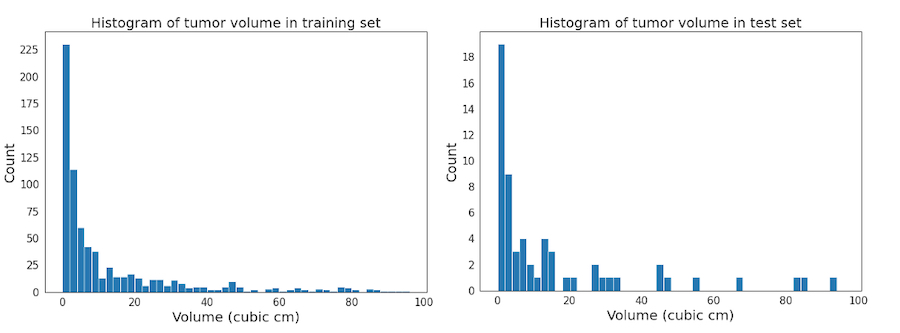

Supplement: Supplementary file 4 — Supplementary Figure 3. [file 41598_2022_19356_MOESM4_ESM.jpg]

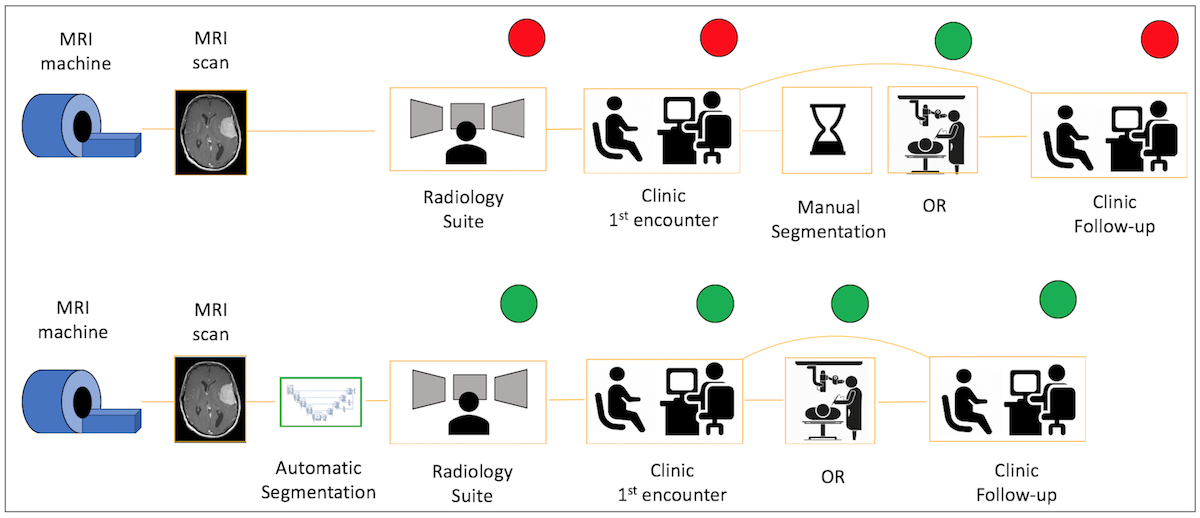

Supplement: Supplementary file 5 — Supplementary Figure 4. [file 41598_2022_19356_MOESM5_ESM.jpg]
