# Supplementary material for: Deep neural networks allow expert-level brain meningioma segmentation and present potential for improvement of clinical practice
Source: Sci Rep. 2022 Sep 14;12:15462. doi: 10.1038/s41598-022-19356-5 (PMC9474556; doi:10.1038/s41598-022-19356-5)
Supplement: Supplementary file 6 — Supplementary Table 1. [file 41598_2022_19356_MOESM6_ESM.docx]

**Supplementary Table 1. Accuracy measures between algorithm predictions and experts’ segmentations**. The table includes accuracy measures (Dice scores) for each MRI in the test set, used to calculate mean and median accuracy measures between algorithm predictions (**Prediction**) and ground truth (**Ground**), and to evaluate inter-expert variability between clinical experts (**Expert_1, Expert_2, Expert_3**) and ground truth and within clinical experts.
