# Supplementary material for: Deep neural networks allow expert-level brain meningioma segmentation and present potential for improvement of clinical practice
Source: Sci Rep. 2022 Sep 14;12:15462. doi: 10.1038/s41598-022-19356-5 (PMC9474556; doi:10.1038/s41598-022-19356-5)
Supplement: Supplementary file 8 — Supplementary Table 3. [file 41598_2022_19356_MOESM8_ESM.docx]

**Supplementary Table 3. Tumor volume values**. Tumor volume values (in cc) for each MRI in the test set, calculated for ground truth (**Ground**), algorithm prediction (**Predicted**), 2D and 3D estimation techniques (**2D estimation, 3D estimation**), clinical experts’ segmentations (**Expert_1, Expert_2, Expert_3**). These measures were used to evaluate differences in tumor volume calculation between algorithm, clinical experts and estimation techniques.
