# Supplementary material for: Deep neural networks allow expert-level brain meningioma segmentation and present potential for improvement of clinical practice
Source: Sci Rep. 2022 Sep 14;12:15462. doi: 10.1038/s41598-022-19356-5 (PMC9474556; doi:10.1038/s41598-022-19356-5)
Supplement: Supplementary file 9 — Supplementary Table 4. [file 41598_2022_19356_MOESM9_ESM.docx]

**Supplementary Table 4. Average absolute tumor volume differences between techniques**. The table presents the average tumor volume differences between ground truth and respectively the model output, expert manual segmentations (**Expert_1, Expert_2, Expert_3**) and 2D/3D volume estimation techniques (**2D estimation, 3D estimation**).

| Ground truth | Average absolute volume difference (cc) | | |
| --- | --- | --- | --- |
| VS | All tumors | >=2cc tumors | <2cc tumors |
|  |  |  |  |
| Model output | 1.90 | 2.66 | 0.27 |
|  |  |  |  |
| Expert_1 | 2.27 | 3.17 | 0.32 |
| Expert_2 | 1.01 | 1.43 | 0.08 |
| Expert_3 | 1.12 | 1.52 | 0.26 |
|  |  |  |  |
| 2D estimation | 10.72 | 15.44 | 0.53 |
| 3D estimation | 6.62 | 9.54 | 0.33 |
